# Unilateral Hypopyon as the First Sign of Relapse in Acute Lymphoblastic Leukemia

**DOI:** 10.3390/hematolrep18050076

**Published:** 2026-10-13

**Authors:** Rasmus Törnroos, Erik Ahlstrand

**Affiliations:** 1 Department of Medicine, Örebro University Hospital, 70185 Örebro, Sweden; rasmus.tornroos@regionorebrolan.se; 2 Department of Medicine, Faculty of Medicine and Health, Örebro University, 70382 Örebro, Sweden

**Keywords:** Ph-B-ALL, ALLTogether (ALL2G), extramedullary relapse, hypopyon, masquerade syndrome, intravitreous metothrexate

## Abstract

**Background and Clinical Significance:** Central nervous system involvement in Acute lymphoblastic leukemia (ALL) is well-documented; however, isolated extramedullary relapse with ocular manifestations is exceedingly rare in adults. Such cases typically present as neoplastic masquerade syndrome, where malignant cells mimic benign intraocular inflammation, often leading to diagnostic delays. In this report, we describe a case of malignant unilateral hypopyon presenting as the sole manifestation of relapse while the patient was in complete morphological bone marrow remission with negative cerebrospinal fluid (CSF) cytology. **Case Presentation:** A 28-year-old male with Philadelphia-negative B-ALL achieved morphological remission using the ALLTogether protocol. During consolidation therapy, he presented with blurred vision that was dependent on postural changes, specifically when bending forward. Ophthalmological examination revealed signs of uveitis and a unique, shifting hypopyon in the left eye. While bone marrow aspirate and CSF cytology remained negative for blasts, flow cytometry of the aqueous humor confirmed an isolated leukemic relapse. Therapeutic management involved escalated systemic chemotherapy, intrathecal injections, and eleven bilateral intravitreal methotrexate injections. Following the achievement of deep molecular remission, the patient underwent allogeneic stem cell transplantation and remains in remission after three years of follow-up. **Conclusions:** Ocular involvement can be a subtle yet critical sign of ALL relapse, even in the absence of detectable systemic disease. This case highlights the importance of maintaining a high index of clinical suspicion for ocular symptoms as potential indicators of isolated relapse, even when standard bone marrow and CSF surveillance suggest complete remission. Furthermore, this report supports the use of intravitreal methotrexate as an effective local adjunct to systemic therapy for achieving optimal disease control.

## 1. Introduction

While central nervous system (CNS) involvement in Philadelphia chromosome-negative B-cell acute lymphoblastic leukemia (Ph-B-ALL) is relatively common, extramedullary relapse with ocular manifestations remains rare in adults [1]. Ocular involvement is typically secondary to confirmed systemic or concurrent CNS disease; however, it can occasionally be the presenting sign of primary leukemia or relapse [2,3,4,5,6]. We report a rare case of an isolated extramedullary ocular relapse in a 28-year-old male with Ph-B-ALL who, despite maintaining complete morphological remission, presented with a unilateral hypopyon and uveitis that initially mimicked benign intraocular inflammation.

## 2. Case Presentation

### 2.1. Presentation and Diagnosis

In April 2022, a 28-year-old Swedish male presented to the emergency department with a one-week history of flu-like symptoms, including cough and fever, followed by jaundice and nausea. Laboratory evaluation revealed elevated liver enzymes and significant hyperbilirubinemia, alongside pancytopenia. Acute viral hepatitis with reactive bone marrow suppression was initially suspected; however, extensive serological and molecular testing returned negative results for hepatitis A, B, C, and E, as well as for other viral infections including CMV, EBV, parvovirus, adenovirus, and HIV. Flow cytometry of the peripheral blood showed only reactive cells, with no evidence of hematological malignancy. Computed tomography of the neck, chest, and abdomen revealed two insignificantly enlarged cervical lymph nodes and mild splenomegaly. Following the spontaneous improvement of blood cell counts, the patient was discharged to outpatient follow-up. Over the subsequent weeks, liver enzymes and bilirubin levels normalized, and the pancytopenia resolved.

In late May, the patient presented with a recurrence of fever and cough. Laboratory investigations confirmed recurrent pancytopenia. A bone marrow aspiration revealed 80% blasts with a BCR-ABL-negative B-ALL phenotype. Although conventional cytogenetics and FISH were normal, a molecular karyotype via microarray revealed deletions of 7p, 9p, and 12q. No recurrent ALL translocations—such as t(2;19), t(17;19), t(12;21), KMT2A rearrangement, or ABL-class fusions—were detected. Minimal residual disease (MRD) marker screening identified two rearrangements in the TCRG gene (VG2a/JG1.1–2.1 and VG2b/JG1.1–2.1), which were subsequently used for disease monitoring via RQ-PCR (limit of detection: 10^−5^). Morphological analysis of the cerebrospinal fluid (CSF) showed isolated blast cells, and the CNS status was classified as CNS2 according to the ALLTogether (ALL2G) protocol—a standardized treatment regimen for ALL patients up to 45 years of age (ClinicalTrials.gov identifier: NCT04307576). Consequently, the final diagnosis was established as Ph-negative B-ALL without formal CNS involvement.

Induction therapy was initiated according to the ALLTogether (ALL2G) protocol, taking into account baseline disease characteristics including CNS2 status. Following Induction B (dexamethasone, vincristine, daunorubicin, peg-asparaginase, and weekly intrathecal methotrexate until two consecutive negative CSF samples were confirmed), complete morphological remission was achieved by Day 29 post-therapy initiation. At this point, minimal residual disease (MRD) analysis was negative by flow cytometry (0.06%), but remained positive via molecular MRD testing (0.2%). Repeated cytological and flow cytometry analyses of the CSF were negative for blasts. Consequently, consolidation treatment proceeded according to the IR-High arm of the ALLTogether protocol (dexamethasone, vincristine, 6-mercaptopurine, cyclophosphamide, cytarabine, and intrathecal methotrexate).

### 2.2. Relapse

Upon admission for Consolidation III, on Day 218 post-therapy initiation, the patient reported blurred vision, discomfort, and redness in the left eye. A critical anamnestic clue was that his vision became severely blurred specifically when bending forward, such as when tying his shoes. This visual impairment was transient, resolving upon returning to an upright position. On repeated ophthalmological examination, visual acuity in the left eye was 1.0 (Snellen) while upright. To investigate the postural dependency of this visual obstruction, the patient was instructed to maintain a downward gaze for 20 s. Following this maneuver, the patient was temporarily unable to read the previously clear line on the eye chart. This visual deficit resolved spontaneously within 15 s after the patient resumed an upright position.

Slit-lamp microscopy of the left eye revealed a ciliary flush in the lower nasal quadrant and a significant hypopyon in the anterior chamber. By tilting the patient’s head to the left, the mobility of the hypopyon was confirmed, suggesting an explanation for the peculiar “shifting” nature of the patient’s blurred vision. Slit-lamp examination also noted heavy flare and the presence of cells in the aqueous humor, though there were no signs of vitreous involvement. While the anterior segment showed marked inflammation, the fundoscopic examination was unremarkable. Due to the presence of a shifting hypopyon, Behçet’s disease was initially included in the differential diagnosis; however, the absence of recurrent oral or genital aphthous ulcers and other characteristic systemic manifestations made the diagnosis less likely. Given the patient’s history of leukemia, an ocular manifestation of ALL was considered the primary etiology. The diagnosis was subsequently confirmed via anterior chamber paracentesis, where flow cytometry of the aqueous humor identified lymphoblasts matching the original B-ALL clone. Crucially, at this time, the bone marrow remained in morphological remission, and the CSF was negative for blasts by both cytology and flow cytometry. MRD analysis on bone marrow aspirate was measured at <0.1% by flow cytometry; unfortunately, molecular MRD testing was not evaluated at this timepoint. A repeated MRI of the brain and spine was also unremarkable, confirming the hypopyon as the sole manifestation of an isolated extramedullary CNS relapse.

### 2.3. Relapse Management

To address the ALL relapse, therapy was escalated to a CNS-directed regimen, and the patient was prepared for a subsequent allogeneic hematopoietic stem cell transplantation as salvage therapy. Systemic treatment was initiated according to Block B1 of the ALL2G protocol, comprising high-dose methotrexate, vincristine, dexamethasone, high-dose cytarabine, and peg-asparaginase. Following a multidisciplinary clinical discussion, intravitreal methotrexate was initiated as a local intervention to target the isolated ocular relapse. The patient received bilateral injections into the vitreous body, with each dose comprising 400 µg of methotrexate diluted in 0.05 mL of 0.9% sodium chloride. A total of eleven bilateral injections were administered. Although the protocol initially mandated twice-weekly administration, the actual frequency was clinically adjusted. A safety threshold was established to withhold injections if the absolute neutrophil count (ANC) fell below 1.0 × 10^9^/L. Concurrently, CNS-directed therapy was augmented with eight intrathecal injections, alternating between methotrexate monotherapy and triple therapy (methotrexate, cytarabine, and dexamethasone). Prior to the relapse, the patient had already received a total of 13 intrathecal methotrexate injections.

Following the initiation of relapse treatment, the patient’s ocular symptoms rapidly resolved, with the exception of transient ocular irritation in the days immediately following intravitreal administration.

After two cycles of Block B1, evaluation confirmed clear CSF, and bone marrow examination showed complete morphological remission, although molecular MRD was still detectable at 0.008%. The patient was subsequently given one cycle of continuous blinatumomab infusion, which successfully induced molecular MRD negativity. After a conditioning regimen comprising total body irradiation, etoposide, and antithymocyte globulin, the patient underwent allogeneic stem cell transplantation with an unrelated donor. Post-transplant, the clinical course was complicated by transient acute graft-versus-host disease; however, the patient remains in MRD-negative complete remission at three years of follow-up.

## 3. Discussion

The occurrence of isolated extramedullary relapse of B-ALL in the eye is a rare clinical event in adults and is reported only in a limited number of cases [1,7,8,9,10]. While the central nervous system is a well-known sanctuary site for leukemic cells, ocular involvement typically manifests alongside active systemic disease or concurrent CNS involvement. This case is notable as a unilateral leukemic hypopyon served as the sole manifestation of relapse, occurring while the bone marrow remained in remission and the CSF was negative for blasts.

The diagnostic journey highlights several clinical challenges. The patient’s initial presentation—featuring jaundice, elevated liver enzymes, and pancytopenia—mimicked acute viral hepatitis. This underscores the importance of maintaining a high index of clinical suspicion for hematological malignancies when pancytopenia is accompanied by atypical inflammatory symptoms. Moreover, the clinical presentation of the ALL relapse highlights the necessity of maintaining a high index of clinical suspicion for ‘masqueraders’ when atypical symptoms arise during consolidation treatment. The term neoplastic masquerade syndrome describes oncological conditions that clinically mimic benign intraocular inflammation [2]. In adults, primary vitreoretinal lymphoma represents the most common cause of this syndrome. Because these malignancies can present with symptoms identical to uveitis, they frequently lead to misdiagnoses and diagnostic delays. Although leukemic infiltration is a significantly less frequent cause of neoplastic masquerade syndrome than vitreoretinal lymphoma, this case demonstrates that it must remain a key differential diagnosis in patients with a history of hematological malignancy. In the Swedish national clinical practice guidelines for ALL, there is no separate, standalone guideline for ophthalmological follow-up that is not related to treatment complications. Cases such as this highlight the need for individualized follow-up when atypical symptoms present during initial presentation or during the clinical course of treatment.

A defining feature of this case was the “shifting” hypopyon. A hypopyon that changes position based on the patient’s head orientation is a rare finding, classically associated with inflammatory Behçet’s disease. Recognizing this atypical presentation allowed for prompt intervention through anterior chamber paracentesis and flow cytometry analysis, which definitively established the leukemic nature of the inflammation.

The management of ocular neoplastic syndromes remains challenging because the eye acts as a pharmacological sanctuary site where systemic chemotherapy often fails to reach therapeutic concentrations. To overcome this barrier, local targeted therapy is required. The decision to utilize intravitreal methotrexate as a rescue therapy was based on available literature and proven clinical experience for similar rare presentations. Intravitreal methotrexate is a well-documented treatment for managing neoplastic masquerade syndromes, particularly in primary vitreoretinal lymphoma [11,12], and its efficacy in leukemic ocular infiltration has also been described [13,14]. In this case, intravitreal methotrexate was successfully adapted for leukemic infiltration, serving as an essential complement to escalated systemic treatment and intrathecal injections. This combined-modality approach led to a rapid resolution of ocular symptoms and facilitated the deep molecular remission necessary for successful allogeneic stem cell transplantation.

In conclusion, ocular involvement in adult ALL may represent a silent but critical manifestation of relapse. This case underscores that any concerning ophthalmological symptoms of intraocular inflammation in a patient with a history of leukemia should be investigated as a potential neoplastic masquerade syndrome. Early recognition of atypical symptoms may facilitate timely intervention and thus potentially improve patient outcomes.

## Data Availability

In this case report, only anecdotal data from medical records were used. The original contributions presented in this study are included in the article.

## Abbreviations

The following abbreviations are used in this manuscript:

CNS	Central Nervous System
Ph-B-ALL	Philadelphia Chromosome-negative B-Cell Acute Lymphoblastic Leukemia
ALL	Acute Lymphoblastic Leukemia
FISH	Fluorescence In Situ Hybridization
CSF	Cerebrospinal Fluid
MRD	Minimal Residual Disease
